# Alarmin S100A8 Activates Alveolar Epithelial Cells in the Context of Acute Lung Injury in a TLR4-Dependent Manner

**DOI:** 10.3389/fimmu.2017.01493

**Published:** 2017-11-13

**Authors:** Deblina Chakraborty, Stefanie Zenker, Jan Rossaint, Anna Hölscher, Michele Pohlen, Alexander Zarbock, Johannes Roth, Thomas Vogl

**Affiliations:** ^1^Institute of Immunology, University of Münster, Münster, Germany; ^2^Department of Anaesthesiology and Intensive Care, University of Münster, Münster, Germany

**Keywords:** damage-associated molecular patterns, S100A8, TLR4, type I alveolar epithelial cells, type II alveolar epithelial cells, IL-6, neutrophils

## Abstract

Alveolar epithelial cells (AECs) are an essential part of the respiratory barrier in lungs for gas exchange and protection against pathogens. Damage to AECs occurs during lung injury and PAMPs/DAMPs have been shown to activate AECs. However, their interplay as well as the mechanism of AECs’ activation especially by the alarmin S100A8/A9 is unknown. Thus, our aim was to study the mechanism of activation of AECs (type I and type II) by S100A8 and/or lipopolysaccharide (LPS) and to understand the role of endogenous S100A8/A9 in neutrophil recruitment in the lung. For our studies, we modified a previous protocol for isolation and culturing of murine AECs. Next, we stimulated the cells with S100A8 and/or LPS and analyzed cytokine/chemokine release. We also analyzed the contribution of the known S100-receptors TLR4 and RAGE in AEC activation. In a murine model of lung injury, we investigated the role of S100A8/A9 in neutrophil recruitment to lungs. S100A8 activates type I and type II cells in a dose- and time-dependent manner which could be quantified by the release of IL-6, KC, and MCP-1. We here clearly demonstrate that AEC s are activated by S100A8 *via* a TLR4-dependent pathway. Surprisingly, RAGE, albeit mainly expressed in lung tissue, plays no role. Additionally, we show that S100A8/A9 is an essential factor for neutrophil recruitment to lungs. We, therefore, conclude that S100A8 promotes acute lung injury *via* Toll-like receptor 4-dependent activation of AECs.

## Introduction

Acute lung injury (ALI) and especially the severe form acute respiratory distress syndrome (ARDS) is a life-threatening disease which encompasses severe lung inflammation leading to respiratory failure and is characterized by alveolar capillary barrier damage (1).

Alveolar capillary barrier is formed of epithelium, interstitial space, and endothelium (2). The epithelium is mainly composed of two cell types. Alveolar epithelial type I cells (AECI) are large squamous cells which help in gas exchange (3, 4) and are renewed by differentiation of AECII to AECI (4). Alveolar epithelial type II cells (AECII) are small cuboidal cells (3) which produce pulmonary surfactant, a major factor to reduce the surface tension of the lung (4). When AECI are damaged during ALI, gas exchange is hampered (5). AECII injury causes decrease in surfactant production which leads to reduced lung compliance and impaired replacement of damaged AECI. AECII has also been shown to differentiate into AECI during culture on fibronectin-coated plates for several days (6).

In the present study, we focused on the role of the endogenous damage-associated molecular patterns (DAMPs) protein S100A8 in combination with the pathogen-associated molecular patterns (PAMPs) molecule lipopolysaccharide (LPS) in initiation of lung inflammation. DAMPs are molecules which act as “alarmins” or warning signals to the body and augment host immune defenses during infection or tissue injury. They are either released passively by necrotic cells during injury/infection or secreted by activated immune cells (7) and help in phagocyte recruitment and activation. During bacterial infection, PAMPs like LPS are present in the alveoli, activating alveolar macrophages which in turn release DAMPs followed by further activation of epithelial and later endothelial cells.

S100A8 and S100A9 [myeloid-related proteins 8 (MRP8) and 14 (MRP14)] represent major DAMPs released during lung inflammation (8, 9). S100A8 and S100A9 homodimers as well as heterodimers are described in the literature (10–12). The S100A8/A9 heterodimer is the most stable and physiologically relevant form (13–15), although for murine S100A8 and S100A9 homodimers, proinflammatory activities in murine models of diseases have been described also (16, 17).

Systemic as well as local levels of S100A8 and S100A9 are increased in respiratory diseases (18). S100A8/A9 also activates alveolar epithelial cells (AECs) (19), which play an important role in maintaining the integrity of alveolar-capillary barrier. However, the mechanism of AEC activation by S100A8/A9 is not yet clear. S100A8 and S100A9 have been shown to bind to the pathogen recognition receptors TLR4 and RAGE (16, 20) and both receptors have been shown to be involved in development of lung injury (21, 22). However, which receptor is mainly involved in S100 mediated AEC activation is still unclear.

Using cell culture assays, murine models of lung inflammation and specific knock-out strains, we now demonstrate that S100A8 drives lung inflammation *via* activation of AECs in a TLR4-dependent manner.

## Materials and Methods

### Materials

The 8- to 10-week-old C57BL/6 WT, S100A9 knockout (S100A9 KO), TLR4 knock out (TLR4 KO), and RAGE knock out (RAGE KO) mice for *in vitro* and *in vivo* experiments were obtained from our animal facility. S100A9 KO mice, TLR4 KO mice, RAGE KO mice, all developed on a C57BL/6 background, were generated as described previously (23–25). To generate TLR4 KO mice, the genomic fragment on chromosome 4 encoding the transmembrane and cytoplasmic domains was replaced with a neomycin resistance gene. To generate RAGE KO mice, the genomic fragment on chromosome 6 encoding the extracellular domain of RAGE was flanked with two loxP sites, which was subsequently deleted on exposure to Cre recombinase. Mice heterozygous for Cre recombination were cross-bred to generate homozygous RAGE KO mice. All animal experiments were approved by the local ethics committee and performed in strict accordance with the German regulations of the Society for Laboratory Animal Science (GV-SOLAS) and the European Health Law of the Federation of Laboratory Animal Science Associations. The protocols were approved by the Landesamt für Natur, Umwelt und Verbraucherschutz Nordrhein-Westfalen, Germany.

Dispase was from BD Biosciences (Heidelberg, Germany). DMEM was from Biochrome (Berlin, Germany). Ultrapure LPS from *Salmonella enterica*, Polymyxin B, DNase I, fibronectin from human plasma, and low melting agarose were from Sigma-Aldrich (Munich, Germany). Airway epithelial media (AEM), a serum free media optimized for airway epithelial cells, along with the following supplements added at indicated final concentrations to AEM—bovine pituitary extract (0.004 ml ml^−1^), epidermal growth factor (10 ng ml^−1^), insulin (5 µg ml^−1^), hydrocortisone (0.5 µg ml^−1^), epinephrine (0.5 µg ml^−1^), triiodo-l-thyronine (6.7 ng ml^−1^), transferrin (10 µg ml^−1^), and retinoic acid (0.1 ng ml^−1^) were from PromoCell GmbH (Heidelberg, Germany). Nylon mesh (28 µm) was from Ted Pella, Inc. (CA, USA). Biotinylated rat antimouse CD45 and CD16/32 antibodies were from BD PharMingen (Heidelberg, Germany). Streptavidin-coated magnetic particles were from Promega (Mannheim, Germany). Rat CD16-32-FITC antimouse was from BD Biosciences (Heidelberg, Germany). Rat CD45-PE/Cy7 antimouse were from Biolegend (Fell, Germany). Rabbit Caveolin-1 and rat CD16-32-FITC antimouse were from BD Biosciences (Heidelberg, Germany). Rat CD 34-FITC antimouse and rat EpCAM-APC antimouse were from eBioscience (Frankfurt, Germany). Hamster T1α antimouse was from Novus Biologicals (CO, USA). Rabbit prosurfactant protein C (proSP-C) antimouse, rabbit RAGE antimouse, and rat TLR4-MD-2 (MTS510) biotin antimouse were from Abcam (Cambridge, UK). Goat Alexa Fluor 488 antirabbit was from Life technologies (OR, USA). Alexa Fluor 488 Streptavidin was from Invitrogen (MA, USA). Goat FITC antirabbit, goat Texas red antihamster, and goat Texas red antirabbit were from Dianova (Hamburg, Germany). Carbon capsule and HEPA capsule were from Pall Corporation (PA, USA). Nebulizers were from Omron MicroAir (Amazon.de, Germany). Cylindrical chambers of Pyrex glass were manufactured by Feinmechanische Werkstatt (Physikalisches Institut, Muenster, Germany).

### Expression and Purification of Murine S100A8 Protein

Murine S100A8 protein was expressed in *Escherichia coli* BL21 (DE3) cells and purified as described previously (16, 26–28). Briefly, murine S100A8 cDNA was cloned into the pET11/20 expression vector and expressed in *E. coli* BL21 (DE3) cells. The bacteria were grown at 37°C in 2× YT. After 24 h, bacteria were harvested, lysed, and inclusion bodies were isolated from the lysates. The inclusion bodies were dissolved in 8 M urea buffer and the sample was adjusted to a pH of 2.0–2.5 by adding hydrochloric acid. In order to establish proper refolding of the protein, samples were then incubated for 60 min at RT and dialyzed stepwise to adapt them to a pH of 7.4 to facilitate refolding in the presence of 2 mM DTT. Samples were then centrifuged at 60,000*g* for 10 min at 4°C to pellet the aggregated material, and further dialyzed before subjecting to anion exchange column and gel filtration chromatography. Protein concentrations were determined by UV absorption at 280 nm using a specific absorption coefficient of 0.40 (mg ml)^−1^ cm^−1^. Limulus amebocyte lysate assay (BioWhitaker, Walkersville, MD, USA) was performed to identify possible endotoxin contamination, which was quantified to 3.6 pg LPS/μg S100A8.

### Isolation of Lung AECs

A method modified from a previous protocol (29) was used for isolation of primary lung AECs. The 8- to 10-week-old WT, TLR4 KO, and RAGE KO mice were anesthetized by ketamine/xylazine intraperitoneally according to body weight. When asleep, the mice were fixed and ~10 ml of 0.9% sodium chloride solution was injected through the right ventricle of the heart. The skin from rib cage to the neck was cut to make the trachea visible. A 22G catheter was inserted into the trachea and 1 ml Dispase (5,000 U) was then injected followed by 0.4–0.5 ml of 1% low melt agarose (stored in a 45°C water bath). Lungs were covered with ice for 2–3 min and extracted out and incubated in 2 ml Dispase. The time and temperature for Dispase incubation was optimized, as described in the result section. The lungs incubated in Dispase were then dissected gently by forceps in a Petri-dish containing 7 ml of DMEM media which was supplemented with 25 mM HEPES buffer, 1% glutamine, 1% penicillin/streptomycin, and 0.01% DNase I. The single cell suspension was passed subsequently through 100 and 40 µm strainers followed by passage through a 28 µm nylon mesh. The filtrate was then centrifuged at 130*g* for 8 min at RT and treated by erythrocyte lysis buffer (155 mM ammonium chloride, 10 mM potassium hydrogen carbonate, and 0.012 mM EDTA in deionized water) to get rid of the erythrocytes, followed by magnetic separation and incubation on Petri-dishes as described in the result section.

### Culture of Primary AECs

The day of isolation was considered day 0. Primary AECs were cultured on fibronectin-coated 24-well plates for 7 days at a density of 0.25–0.35 × 10^6^ cells/well to differentiate into type I cells. To maintain the type II phenotype, cells were cultured on 24-well plates at a density of 0.9–0.95 × 10^6^ cells/well for 28–30 h.

### Murine Model of Lung Inflammation

The 8- to 12-week-old mice were placed inside a cylindrical Pyrex chamber which was connected to a nebulizer containing a solution of LPS (0.5 mg ml^−1^) or S100A8 (0.28 mg ml^−1^) in saline or only saline. Mice were exposed to LPS or S100A8 or saline vapors for 45 min followed by a resting period of 4 h. After 4 h, mice were anesthetized and fixed on dissecting board and their skin was cut from the abdomen to the throat so as to expose the trachea. Blood was collected from heart to obtain serum for S100A8/A9 analysis by enzyme-linked immunosorbent assay (ELISA). Thereafter, a 22 G catheter was inserted into the trachea. BAL was collected by flushing the lungs five times with 0.8 ml PBS through the trachea and pulling out the BAL from the lungs into the syringe attached to the catheter. The collected BAL was centrifuged at 400*g* for 5 min at RT. The supernatant was collected and stored at −80°C for later use in analyzing cytokine/chemokine expression by ELISA or bead based immunoassays. The cell pellet was counted by Kimura staining (30) to identify the number of polymorphonuclear neutrophils (PMNs) in BAL.

### Flow Cytometry

Flow cytometric staining was done by collecting ~4–5 × 10^5^ cells in FACS tubes followed by treatment with the Fc blocking reagent, anti-CD16-32 antibody, for 10 min at 4°C. Primary antibody (rat anti-EpCAM or rabbit anti-proSP-C) was then added in 1% FBS/PBS for 30 min at 4°C, followed by secondary antibody (anti-ratIgG-APC or antirabbitIgG-FITC) incubation for 30 min at 4°C. For proSP-C staining, intracellular staining was performed wherein cells were permeabilized using BD Cytofix/Cytoperm Plus Fixation/Permeabilization Kit from BD Biosciences (Heidelberg, Germany) before primary antibody addition, according to the manufacturer’s instructions.

### Immunofluorescent Staining of AECs for TLR4-MD-2 and RAGE

Alveolar epithelial cells were seeded on fibronectin-coated 8-well LabTek chambers at a density of ~3 × 10^5^ (AECI) or ~7 × 10^5^ cells (AECII)/well for 7 or 1 day, respectively. Immunofluorescent staining was performed by fixing the cells with 4% paraformaldehyde for 10 min at RT, followed by permeabilization by 0.5% TritonX-100 for 10 min at RT. Cells were then incubated in 10% normal goat serum blocking buffer for 1 h at RT, followed by incubation in primary antibody (rat anti-TLR4-MD-2-biotin or rabbit anti-RAGE) diluted in 0.2% BSA in PBS-TritonX-100, at 4°C overnight. Secondary antibody (streptavidin Dylight 488 or antirabbit Alexa Fluor 488) was then added in 0.2% BSA in PBS-TritonX-100 to the cells. DAPI was then used to stain the nucleus, followed by addition of fluorescent mounting medium (Dako) and visualization by an inverted fluorescent microscope (Axio Observer, Zeiss, Germany).

### IL-6 Analysis by ELISA

Supernatant collected from stimulated AECs was analyzed for IL-6 by murine IL-6 ELISA kit from eBiosciences (Frankfurt, Germany) following manufacturer’s instructions.

### S100A8/A9 and IgM Analysis by ELISA

BAL from WT and S100A9 KO mice, and serum from WT mice were used to analyze S100A8/A9 by ELISA as described in Ref. (31). Also, BAL of WT and S100A9 KO mice were analyzed for IgM by murine IgM ELISA kit from Thermo Fisher Scientific (Dreieich, Germany) following manufacturer’s instructions.

### Cytokine/Chemokine Measurements by Bead Based Immunoassay

Several cytokines/chemokines, namely—MCP-1, GM-CSF, IFN-β, IFN-γ, IL-1α, IL-1β, IL-10, IL-12 (p70), IL-17A, IL-23, IL-27, TNF-α, KC, RANTES, IL-5 were measured by a bead based immunoassay called LEGENDplex, according to manufacturer’s instructions, using the principles of sandwich immunoassays. The mouse inflammation panel, proinflammatory chemokine mix and match panel and custom mouse panel from LEGENDplex (Biolegend, Germany) were used to detect various analytes. Analyte measurements were made using Navios flow cytometer.

### Statistical Analysis

Statistical significance was calculated of three or more experiments by Prism 5 software using either unpaired two-tailed Student’s *t*-test compared to the control or two-way ANOVA (with Bonferroni’s or Tukey’s test) or one-way ANOVA (with Bonferroni’s test) (32, 33). Results are presented as means ± SEM. *p* < 0.05 was considered significant.

## Results

### Optimizing Isolation, Purification, and Culturing of AECs

**Figure 1:**
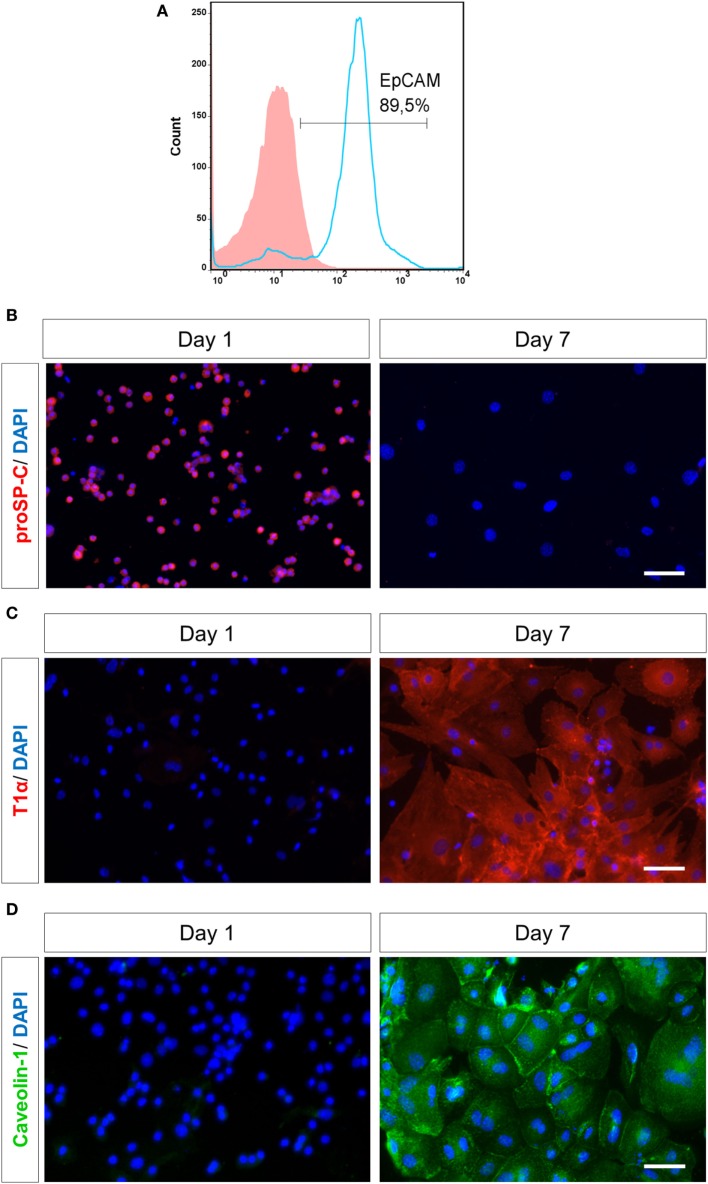
Purity and characterization of murine alveolar epithelial cells (AECs) after isolation. **(A)** Murine lung cells were stained by flow cytometry for EpCAM (APC) after negative magnetic bead purification and plating on Petri-dishes for 4 h. Solid histogram depicts isotype control, open histogram depicts EpCAM staining. EpcAM, epithelial cell adhesion molecule; APC, allophycocyanin. AECs were characterized based on staining for various cell surface markers, like **(B)** AECII-specific marker proSP-C (Texas red), **(C)** AECI-specific marker T1α (Texas red) and **(D)** AECI-specific marker Caveolin-1 (FITC) on day 1 or 7 after seeding. Nuclei were stained with DAPI. Imaging was performed by an inverted fluorescent microscope (Axio Observer, Zeiss). Scale bars are 50 µm. proSP-C, prosurfactant protein C; FITC, fluorescein isothiocyanate.

A previously described protocol for AEC culture and differentiation (29) was modified and optimized to achieve the desired purification and to prevent pre-activation of AECs. The original protocol was first optimized regarding Dispase digestion, i.e., lungs were incubated in 2 ml dispase for 20 min at 37°C, with constant rotation. For the first purification step, cells were subjected to magnetic bead separation, using biotinylated CD45 and CD16/32 antibodies for 30 min at 37°C, followed by streptavidin-coated magnetic beads for 30 min at RT, as described previously (29). However, the amount of DMEM for suspending the cells in antibodies and beads was optimized to 500 µl. Further purification was achieved by plating cells on Petri-dishes to remove adherent mesenchymal cells. Time of plating was optimized to 4 h, whereas a range of 4–16 h was recommended previously (29). Cells were then stained for Epithelial Cell Adhesion Molecule, EpCAM [epithelial cell-specific marker (34)] by flow cytometry, showing a purity of ~90% (Figure 1A). Contaminating leukocytes (CD45/CD16-32/CD34 positive cells) were completely undetectable (Figure S1A in Supplementary Material).

Initially, AECs were cultured in DMEM + 10%FBS as described earlier (29, 35), but they were spontaneously activated when cultured in FBS containing medium. Therefore, AECs were subsequently cultured in a special AEM along with supplements that supported cell growth without FBS addition. AECs cultured in this media showed a significantly reduced spontaneous activation (Figure S1B in Supplementary Material).

Freshly isolated primary AECs were mainly type II cells, as confirmed by analysis for proSP-C (Figure S1C in Supplementary Material), which is the precursor protein of type II-specific marker SPC (36). AECs were cultured on fibronectin-coated culture plates for 7 days to obtain a type I phenotype by differentiation of AECII (6). A type II monolayer was obtained by culturing AECs at a high density for 28–30 h after isolation. The type I cells (AECI) and type II cells (AECII) were characterized based on cell-specific markers (Figures 1B–D). Most AECs were positive for proSP-C on day 1. By day 7, the cells lost proSP-C indicating that they had differentiated, now expressing AECI-specific markers T1α and Caveolin-1, confirming differentiation of AECII to AECI during 7 days in culture.

### S100A8-Induced IL-6 Release from AECs follows a Dose- and Time-Dependent Pattern

**Figure 2:**
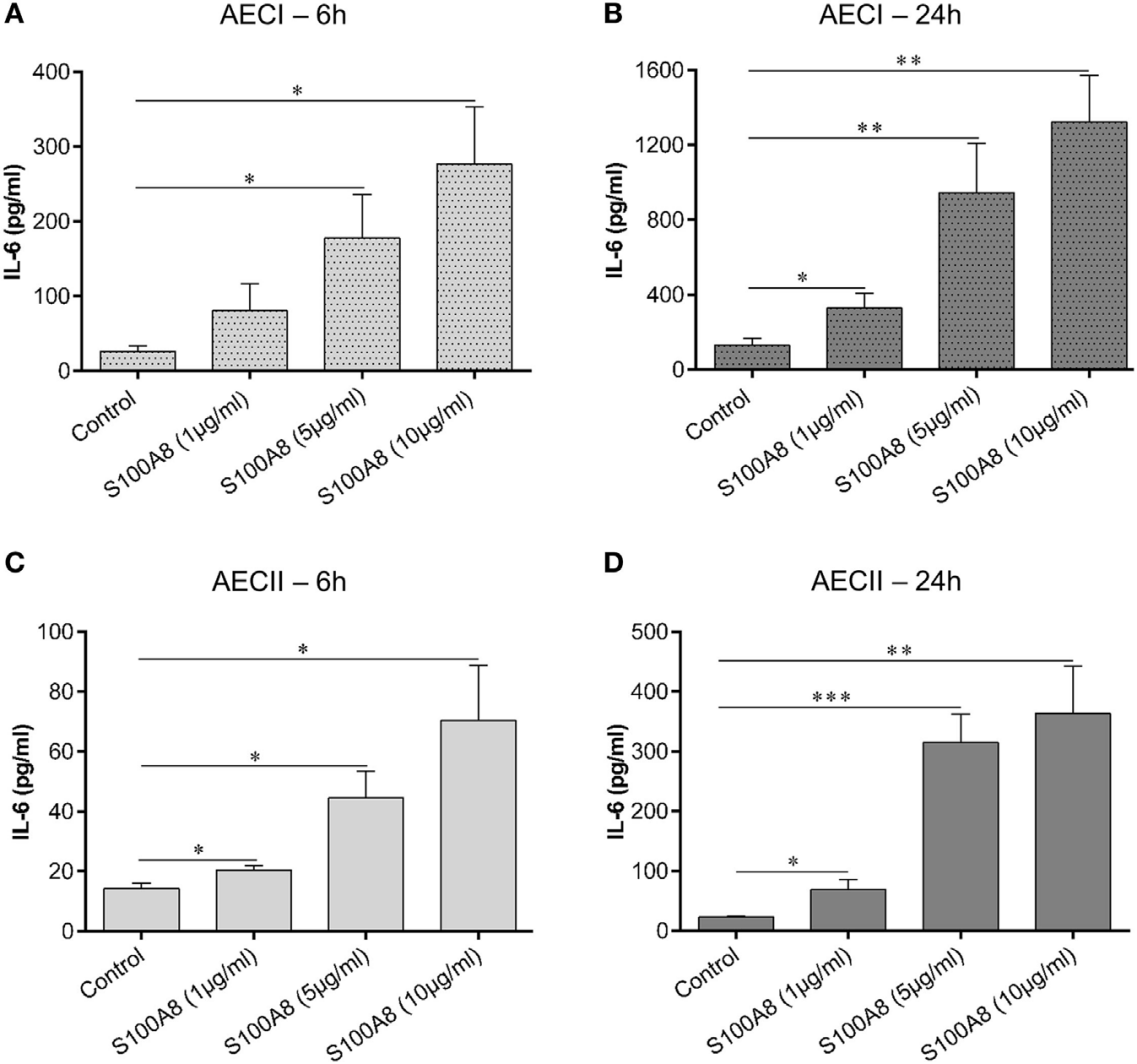
S100A8 activates alveolar epithelial type I cell (AECI) and type II cell (AECII) in a dose and time-dependent manner. AECI and AECII were stimulated for 6 h **(A,C)** and 24 h **(B,D)** with increasing amounts of murine S100A8 as indicated in the figure. The levels of IL-6 as a readout of epithelial cell activation was quantified by enzyme linked immunosorbent assay (ELISA). Data are expressed as mean ± SEM (*n* = 4 experiments). *p < 0.05, **p < 0.01, ***p < 0.001 compared to unstimulated cells (Control).

Alveolar epithelial type I cell (AECI) and type II cell (AECII) were stimulated for 6 or 24 h with increasing concentrations of murine S100A8 as indicated (Figures 2A–D) and IL-6 in the supernatant was measured by ELISA after each time point. For both AECI and AECII, IL-6 release increased proportionally with increasing concentrations of S100A8 at each time point of stimulation. For both AECI and AECII, IL-6 release from stimulated and non-stimulated cells increased over time from 6 to 24 h. Generally, AECI showed a stronger secretion of IL-6 than AECII (6 and 24 h) (Figure 2). The observed effects were not influenced by possible endotoxin contamination due to the low content of 3.6 pg LPS/μg S100A8 in the preparation. Up to 100 pg ml^−1^ LPS did not activate human monocytes (Figure S2A in Supplementary Material) and addition of Polymyxin B (an LPS inhibitor) did not affect the release of TNF-α from S100A8 stimulated monocytes. Human monocytes were isolated and stimulated as described earlier (26). Also, up to 50 pg ml^−1^ LPS did not activate AECI to release IL-6 and addition of Polymyxin B to LPS (10 ng ml^−1^) completely blocked its IL-6 release capacity from AECI (Figure S2B in Supplementary Material). In contrast, addition of Polymyxin B to S100A8 (5 µg ml^−1^) did not affect its IL-6 release capacity from AECI.

### Cytokine/Chemokine Response Induced by S100A8 in AECs Compared to LPS

**Figure 3:**
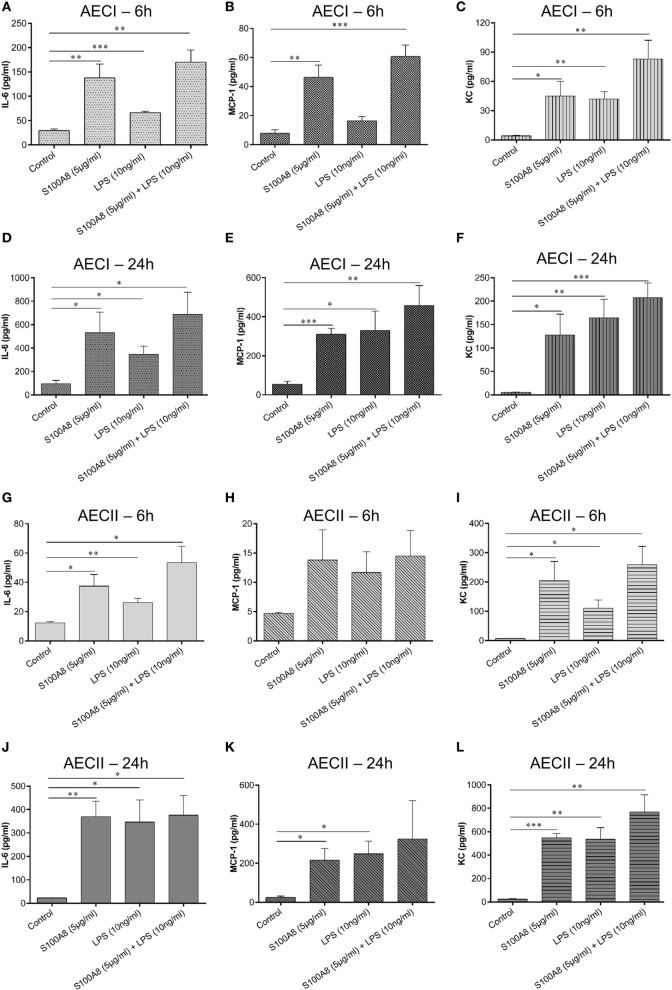
S100A8 and lipopolysaccharide (LPS)-induced production of various cytokines/chemokines from alveolar epithelial cells (AECs). Murine S100A8 (5 µg ml^−1^) or LPS (10 ng ml^−1^) or a combination of both were used to stimulate confluent monolayers of AECI **(A–F)** or AECII **(G–L)**. **(A,D,G,J)** IL-6, **(B,E,H,K)** MCP-1, and **(C,F,I,L)** KC were measured after 6 and 24 h of stimulation. Data are expressed as mean ± SEM [*n* = 4 experiments for AECI **(A–F)**, *n* = 3 experiments for AECII **(G–L)**]. *p < 0.05, **p < 0.01, ***p < 0.001 compared to respective unstimulated cells (controls).

In order to understand the interplay of DAMPs (S100A8) and PAMPs (LPS) in activating epithelial cells layers, confluent monolayers of AECI and AECII were stimulated for 6 and 24 h by murine S100A8 or LPS or a combination of both. Supernatant collected after 6 and 24 h was analyzed by either ELISA or bead based immunoassay for several cytokines/chemokines, namely—GM-CSF, IFN-β, IFN-γ, IL-1α, IL-1β, IL-6, IL-10, IL-12 (p70), IL-17A, IL-23, IL-27, MCP-1, RANTES, TNF-α, and KC. It was observed that AECs released only IL-6, MCP-1, and KC in response to S100A8 or LPS or their combination (Figures 3A–L).

S100A8 caused a significant release of IL-6, MCP-1, and KC from AECI (Figures 3A–C) and AECII (Figures 3G–I) as compared to non-stimulated controls at 6 h. LPS caused a significant release of IL-6 and KC from AECI (Figures 3A,C) and AECII (Figures 3G,I) as compared to non-stimulated controls at 6 h. Release of IL-6/MCP-1/KC induced by combination of S100A8 and LPS was significantly higher than controls and for most conditions, we could observe mainly additive effects after 6 h of stimulation. Although, for some conditions, the cytokine/chemokine release by the combination of S100A8 and LPS did not reach the summary of individual stimulations (S100A8 or LPS), it is clear that there are definitely no competitive effects between S100A8 and LPS stimulation. The release of IL-6/MCP-1/KC after 24 h was similar irrespective of the stimulant used in case of both AECI and AECII (Figures 3D–F and 3J–L).

### Analysis of TLR4-MD-2 and RAGE Expression in AECs

**Figure 4:**
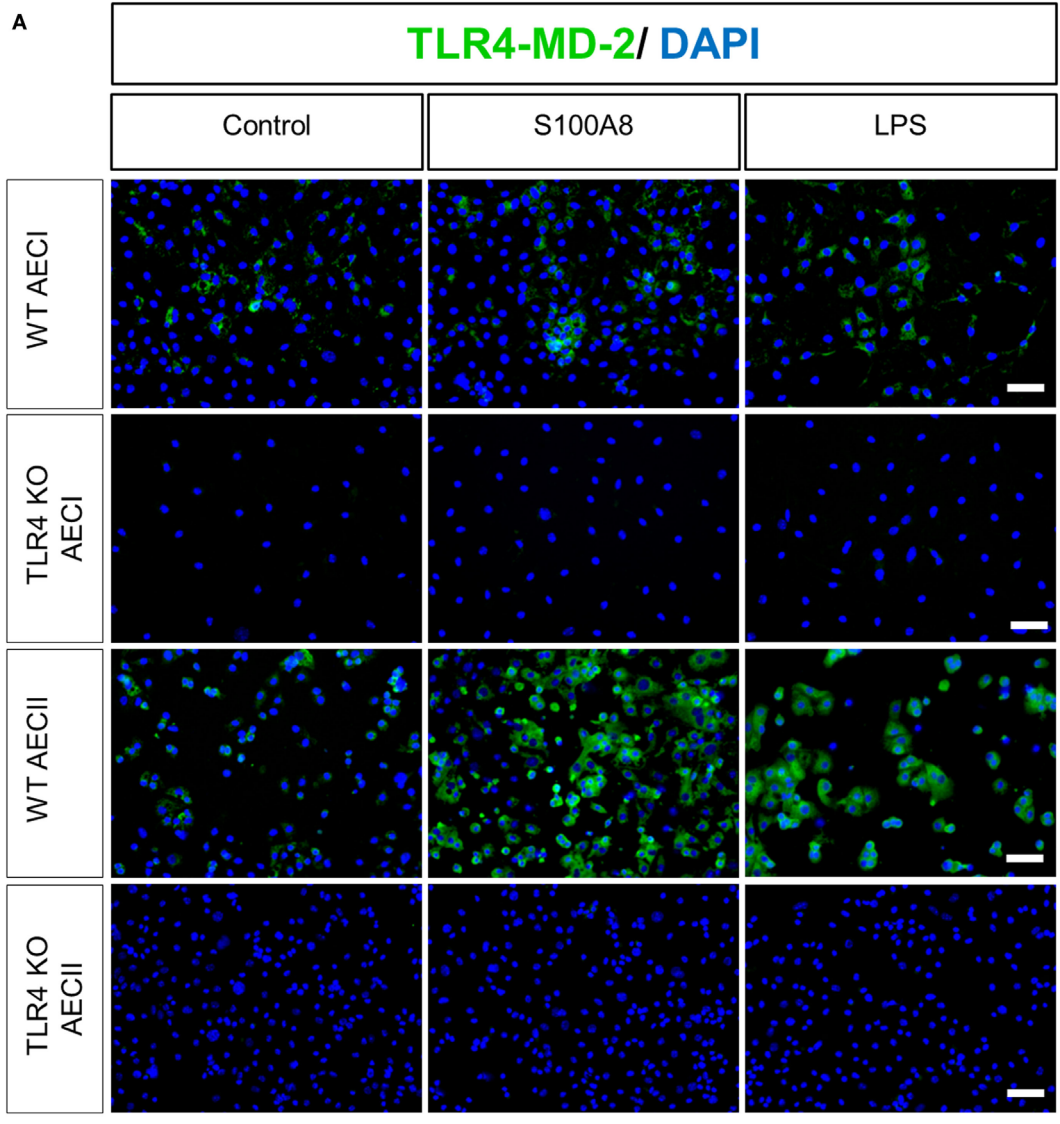

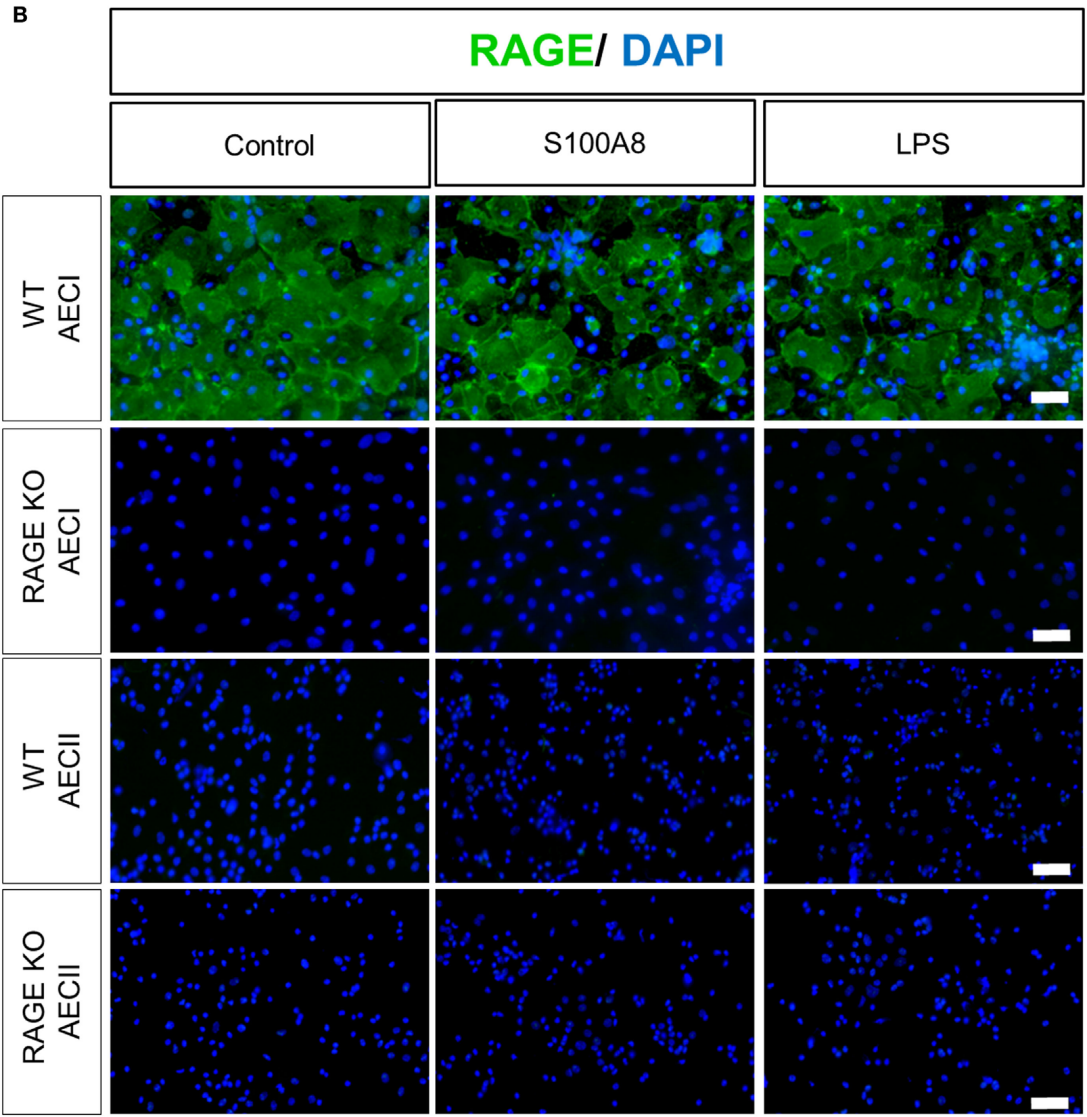
TLR4-MD-2 and RAGE expression in isolated alveolar epithelial cells (AECs). WT, TLR4 knock out (TLR4 KO), and RAGE knock out (RAGE KO) AECII and AECI were cultured for 1 or 7 days, respectively, and then stimulated by murine S100A8 (5 µg ml^−1^) or lipopolysaccharide (LPS) (10 ng ml^−1^) for 6 h. **(A)** WT and TLR4 KO AECI and AECII were stained for TLR4-MD-2 (Streptavidin Dylight 488) **(B)** WT and RAGE KO AECI and AECII were stained for RAGE (goat anti rabbit Alexa Fluor 488). Nuclei were stained with DAPI. Imaging was performed by an inverted fluorescent microscope (Axio Observer, Zeiss), *n* = 3 experiments for **(A,B)** and representative images are shown. Scale bars are 50 µm.

Human AECII had been shown to express very low basal amounts of TLR4, which increased upon stimulation by LPS (37). Based on these observations, WT and TLR4 KO murine AECI and AECII monolayers cultured on fibronectin-coated Labtek chambers were stimulated with murine S100A8 or LPS for 6 h, before staining for TLR4-MD-2 (Figure 4A). WT AECI and AECII showed a low expression of TLR4-MD-2 under non-stimulated conditions. AECI showed no increase in TLR4-MD-2 expression from its basal level, after stimulation. However, expression of TLR4-MD-2 increased markedly in WT murine AECII after stimulation by S100A8 and LPS. To confirm that the increase in the expression of TLR4-MD-2 induced in WT AECII by S100A8 was not due to the presence of endotoxin contamination, we compared the TLR4-MD-2 expression of WT AECII stimulated by S100A8 and LPS to that of WT AECII stimulated by a combination of Polymyxin B and S100A8 or LPS (Figure S3 in Supplementary Material). Increase in TLR4-MD-2 expression in AECII by stimulation with S100A8 + Polymyxin B was identical to S100A8 stimulation alone. However, when Polymyxin B was added to LPS stimulation, the TLR4-MD-2 expression markedly decreased as compared to LPS stimulation alone, and reached basal levels. RAGE expression was examined in AECs by stimulating WT and RAGE KO AECI and AECII with S100A8 or LPS for 6 h and subsequent staining for RAGE (Figure 4B). WT AECI showed a high basal expression of RAGE which did not increase further upon S100A8/LPS stimulation. AECII showed no RAGE expression.

### S100A8 Induces IL-6 Release in AECI and AECII *via* TLR4

**Figure 5:**
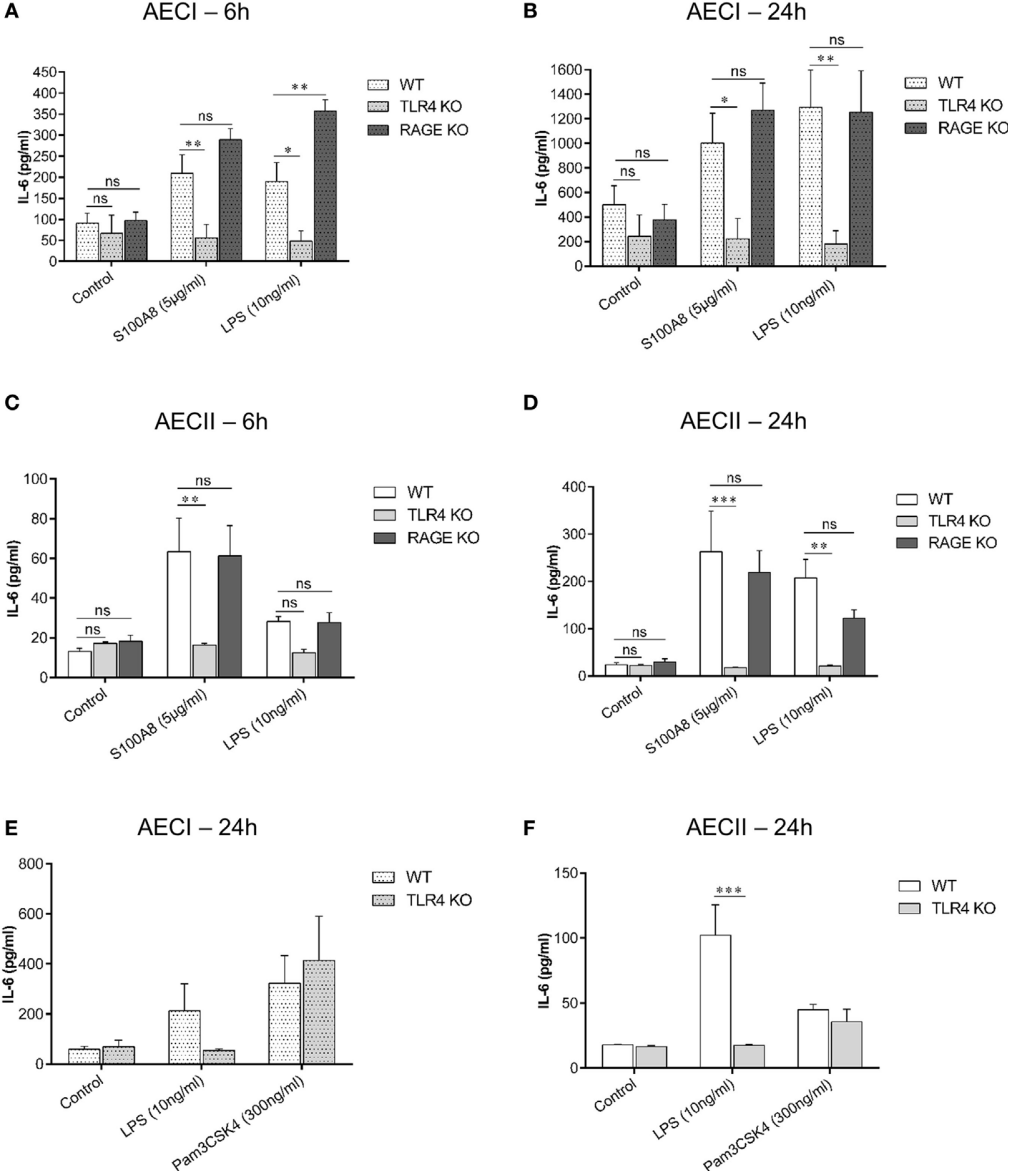
TLR4 signaling pathway is involved in IL-6 release from alveolar epithelial cells (AECs). AECI **(A,B)** and AECII **(C,D)** from WT, TLR4 knock out (TLR4 KO), and RAGE knock out (RAGE KO) mice were stimulated with murine S100A8 (5 µg ml^−1^) or lipopolysaccharide (LPS) (10 ng ml^−1^) for **(A,C)** 6 h and **(B,D)** 24 h. IL-6 levels were analyzed by enzyme linked immunosorbent assay (ELISA). Data are expressed as mean ± SEM (*n* = 3 experiments). *p < 0.05, **p < 0.01, ***p < 0.001, ns, not significant, compared to WT AECs. Confluent monolayers of **(E)** AECI or **(F)** AECII from WT and TLR4 KO mice were stimulated with TLR agonists, LPS or Pam3CSK4 for 24 h and IL-6 levels were analyzed by ELISA. Data are expressed as mean ± SEM [*n* = 3 experiments for AECI **(E)**, *n* = 4 experiments for AECII **(F)**]. ***p < 0.001, compared to WT AECs.

As mentioned earlier, S100A8 binds to TLR4 and RAGE *in vitro*. To explore the role of TLR4 and RAGE in AEC activation, AECs were isolated from WT, TLR4 KO and RAGE KO mice and cultured for 7 days (AECI) and 1 day or 28 h (AECII). Thereafter, AECs were stimulated with murine S100A8 or LPS for 6 and 24 h and supernatants were analyzed for IL-6 by ELISA (Figures 5A–D). At both time points, IL-6 release from S100A8/LPS stimulated RAGE KO AECI was not reduced compared to that from S100A8/LPS stimulated WT AECI, in case of LPS it was even higher in RAGE KO AECI. In contrast, IL-6 release from stimulated TLR4 KO AECI was significantly repressed as compared to that from stimulated WT AECI. IL-6 release from TLR4 KO AECI was similar to that released from non-stimulated controls indicating that in absence of TLR4, AECI were not responding to S100A8 or LPS stimulation. Thus, although AECI expresses RAGE in high amounts as compared to low amounts of TLR4-MD-2 expression, IL-6 release from S100A8 stimulated AECI is TLR4 dependent and independent of RAGE. Accordingly, IL-6 release induced by S100A8, from TLR4 KO AECII was significantly repressed as compared to that from WT AECII at each time point but RAGE KO AECII showed similar levels of IL-6 release compared to WT AECII. LPS stimulated TLR4 KO AECII showed already a lower IL-6 release after 6 h compared to WT AECII which, however, did not reach statistical significance. This trend at 6 h became significant after 24 h stimulation.

In order to prove that the unresponsiveness of TLR4 KO AECs to S100A8 was due to the lack of TLR4 and not due to secondary mechanisms, AECI and AECII from WT and TLR4 KO mice were stimulated with the TLR4 agonist LPS or the TLR1/2 agonist Pam3CSK4 (Figures 5E,F). IL-6 release from Pam3CSK4 stimulated TLR4 KO AECs was similar to that from Pam3CSK4 stimulated WT AECs. Therefore, the lack of TLR4 in AECs has no general effect on the inflammatory response of AECs.

### Absence of Endogenous S100A8/A9 Hampers Neutrophil Recruitment in ALI Affected Murine Lungs

To understand the *in vivo* role of endogenous S100A8/A9 in neutrophil recruitment during ALI, a murine model of ALI was created by exposure to LPS or S100A8 vapors. Saline exposed mice served as controls. For these studies, WT and S100A9 KO C57BL/6 mice were used. S100A9 KO mice lack both S100A8 and S100A9 on the protein level (23, 38), as observed in the western blot of bone marrow cells from WT and S100A9 KO mice (Figure S4 in Supplementary Material). Murine bone marrow cells were isolated as described earlier (16) and western blot was performed in a manner similar to that described in a previous study (26).

**Figure 6:**
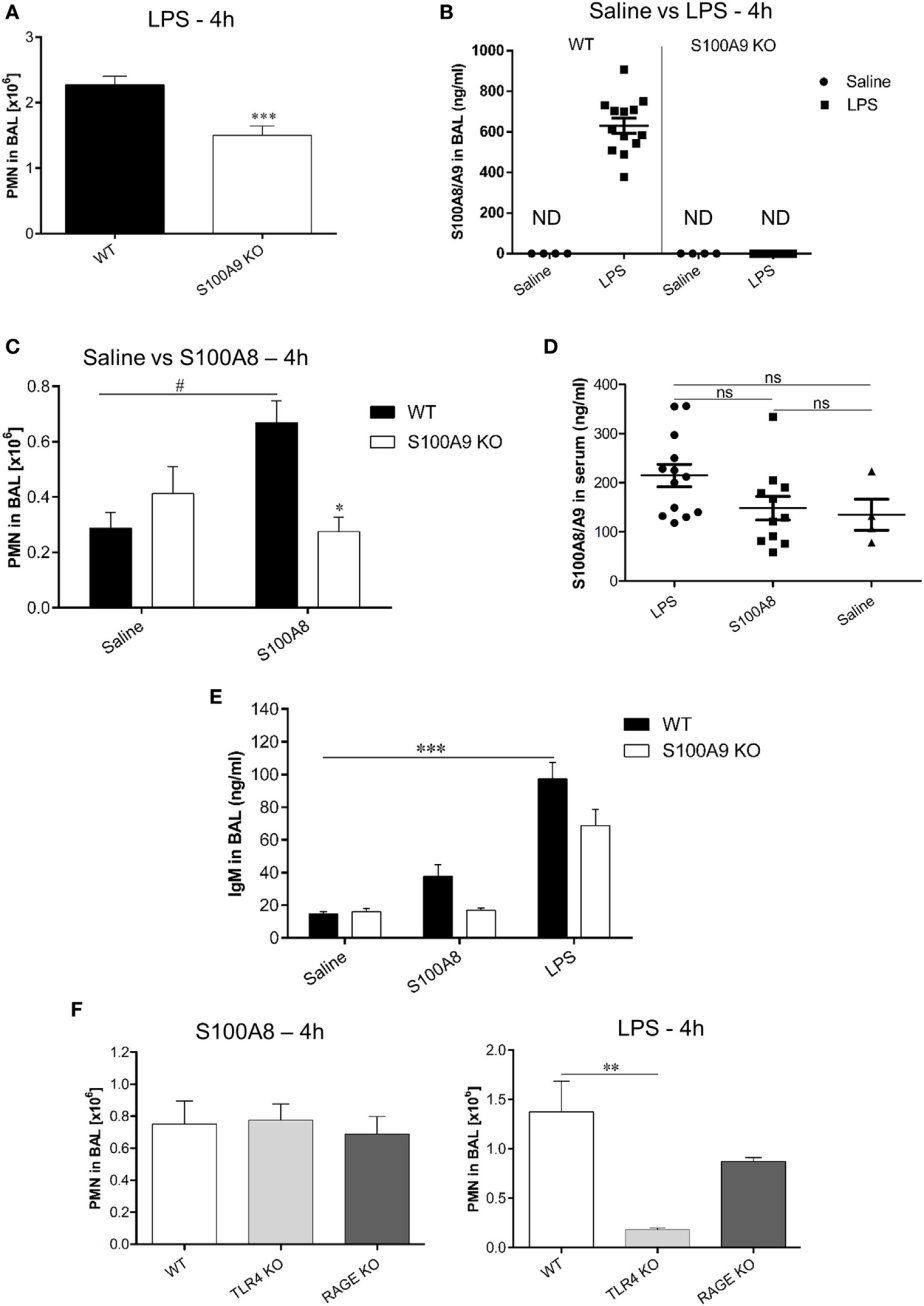
Comparison of polymorphonuclear neutrophil (PMN) recruitment and S100A8/A9 release in BAL and serum of WT and S100A9 KO mice exposed to lipopolysaccharide (LPS) or S100A8. **(A)** WT and S100A9 KO mice were exposed to LPS and PMN were counted in BAL after 4 h. Data are expressed as mean ± SEM (*n* = 11 mice per group). ***p < 0.001 compared to WT mice. **(B)** S100A8/A9 release was analyzed by enzyme linked immunosorbent assay (ELISA) in the BAL of WT or S100A9 KO mice 4 h after saline or LPS inhalation. Data are expressed as mean ± SEM (*n* ≥ 10 mice per group for LPS and *n* = 4 mice per group for saline). ND, not detectable. **(C)** WT and S100A9 KO mice were exposed to saline or murine S100A8 and PMN were counted in BAL after 4 h of exposure. Data are expressed as mean ± SEM (*n* = 4 mice per group for saline and *n* = 6 mice per group for S100A8). *p < 0.01 compared to S100A8 exposed WT mice. ^#^*p* < 0.01 compared to saline exposed WT mice. **(D)** S100A8/A9 release was analyzed by ELISA in serum of WT mice exposed to LPS or S100A8 or saline. Data are expressed as mean ± SEM (*n* = 13 mice per group for LPS, *n* = 11 mice per group for S100A8, and *n* = 4 mice per group for saline exposure). ns, non-significant. **(E)** IgM release was analyzed by ELISA in BAL of WT and S100A9 KO mice 4 h after exposure to saline or murine S100A8 or LPS. Data are expressed as mean ± SEM (*n* = 4 mice per group for saline, *n* = 5 mice per group for S100A8, and *n* = 10 mice per group for LPS). ***p < 0.001compared to saline exposed WT mice. **(F)** WT, TLR4 KO, and RAGE KO mice were exposed to murine S100A8 or LPS and PMN were counted in BAL after 4 h of exposure. Data are expressed as mean ± SEM (*n* = 4 mice per group for LPS and S100A8). **p < 0.01 compared to WT mice.

BAL from LPS exposed WT and S100A9 KO mice was analyzed after 4 h, for PMN counts, which were significantly higher in the BAL of WT mice than in the BAL of S100A9 KO mice (Figure 6A). The reason for higher PMN counts in WT compared to S100A9 KO mice could be because LPS exposure caused a high release of S100A8/A9 (~600 ng ml^−1^) in BAL of WT mice (Figure 6B), which then acted as a chemoattractant for PMNs. LPS exposed S100A9 KO mice, as expected, showed non-detectable S100A8/A9 levels. The strong induction of S100A8/A9 release by LPS could be further confirmed by comparing the high S100A8/A9 levels in LPS-treated WT mice to non-detectable S100A8/A9 levels in untreated (saline treated) WT mice. To confirm that LPS exposure together with the S100A8/A9 release caused PMN infiltration in BAL, we examined the effect of exogenous S100A8 application on PMN counts in BAL of WT mice. We observed that PMN counts of WT mice exposed to S100A8 were higher than saline exposed WT mice (Figure 6C). S100A9 KO mice exposed to S100A8 showed basal levels of PMNs in BAL which was similar to that shown by saline-treated (WT or S100A9 KO) mice and was significantly lower than WT mice exposed to S100A8, confirming that S100A8/A9 is required for PMN infiltration in BAL in response to DAMP/PAMP exposure. Systemic release of S100A8/A9 in LPS or S100A8 exposed WT mice was comparable to saline exposed WT mice (Figure 6D) indicating no systemic involvement of S100A8/A9 in PMN recruitment to BAL at this early time point. PMNs in BAL were also counted 24 h after LPS exposure (data not shown), but the differences between WT and S100A9 KO PMN counts were not significant after 24 h. We also studied the release of IgM in BAL, which is a biomarker of lung injury (Figure 6E). We compared the release of IgM in BAL of WT mice exposed to LPS or S100A8 to that of untreated (saline treated) WT mice. The IgM response in BAL was significantly higher after 4 h of LPS exposure. Although not significant, the IgM response in BAL of S100A8-treated mice was higher than that of untreated mice. Thus, it can be concluded that after 4 h, LPS exposure leads to a strong injury response in BAL of mice and S100A8 exposure leads to a mild injury response in BAL of mice. We also compared the IgM response between LPS/S100A8 exposed WT and S100A9 KO mice and found that IgM response of S100A9 KO mice, although non-significant, was lower than the corresponding LPS/S100A8 exposed WT mice. The role of TLR4 and RAGE receptors in neutrophil recruitment to BAL was also studied, by analyzing BAL from LPS/S100A8 exposed WT, TLR4 KO and RAGE KO mice for PMN counts (Figure 6F). For S100A8 treatment, there was no difference in the PMN counts between the WT, TLR4 KO and RAGE KO mice, indicating that TLR4 and RAGE do not influence neutrophil recruitment *in vivo* during S100A8 exposure. However, for LPS treatment, PMN counts in BAL of TLR4 KO mice were significantly lower than that of WT mice. Although not significant, the PMN counts in BAL of LPS-treated RAGE KO mice were lower than that of WT mice, and thus RAGE could play a role in PMN recruitment to BAL during LPS exposure, independent of S100A8/A9. The mechanism behind this finding is not known and could be analyzed by further investigation.

## Discussion

Pathogen-associated molecular patterns like LPS trigger the release of DAMPs from activated alveolar macrophages in the alveoli. The following infiltrating neutrophils and activated circulating local alveolar macrophages produce cytokines/chemokines, oxidants, proteases, and more DAMPs (39, 40) which in turn may damage alveolar epithelial and endothelial cells in combination with PAMP induced effects (41, 42), thus degrading the alveolar-capillary barrier. This leads to uncontrolled neutrophil infiltration, tissue damage and overwhelming immune responses in ALI. Our current study focused on the mechanisms of activation of AECs by S100A8 in synergy with LPS.

Although the S100A8/A9 heterodimer is the most physiologically relevant form, we used S100A8 homodimer for stimulation experiments because it has been shown that S100A8 is the active component of the heterodimer and activates for, e.g., human as well as murine monocytes (16, 26).

To study the effect of S100A8 on AECs, we modified the common isolation protocol resulting in highly purified AECII (~90%) able to differentiate to AECI without signs of preactivation. Main steps were optimization of time and temperature for Dispase digestion, magnetic bead separation using small volume of media to increase antibody and bead concentration, maintaining of type II phenotype in high density monolayers, and the switch to FBS-free culture conditions that reduced pre- or self-activation of the cells.

S100A8-dependent IL-6 secretion from AECI and AECII increased in a concentration and time-dependent manner. To understand the interplay of DAMPs and PAMPs in activating AECs, LPS, and S100A8 were used together. The cytokine/chemokine repertoire released by AECs was very specific. Out of the many proinflammatory mediators examined, AECI and AECII produced only IL-6, MCP-1, and KC by S100A8 or LPS induction, indicating that S100A8 and LPS induce similar cytokine/chemokine release pathways.

Since RAGE and TLR4 have been shown to be present on AECs (43–45), and S100A8 is able to act as an agonist for both, it was necessary to find the individual contributions of both receptors. To explore this, we first analyzed the expression of the receptors in AECs. Murine AECI showed a high RAGE expression which is in line with a previous study on rats showing that RAGE was present on the basal membrane of AECI but absent on AECII (44). TLR4-MD-2 was present in low amounts on AECI and AECII under non-stimulated conditions. Upon S100A8 stimulation, AECII showed an increase in TLR4-MD-2 expression, similar to what had been observed for LPS stimulation of human AECII (37). TLR4-MD-2 staining was performed instead of TLR4 staining because TLR4 binds its ligand LPS only in form of a TLR4-MD-2 complex and MD-2 is essential for activation (46). Despite the dominant expression of RAGE compared to TLR4, our data clearly show that TLR4 is exclusively responsible for S100A8-mediated effects on AECs and RAGE plays no relevant role in our chosen conditions.

Production of an almost identical pattern of cytokines/chemokines by stimulation of S100A8, LPS, or combined S100A8/LPS stimulation supports the assumption that both stimulants act *via* the TLR4-MD-2 pathway in AECs. We showed that both S100A8 and LPS activate AECs *via* TLR4, but the kinetics of binding to TLR4 may be different in case of both stimuli. We observed additive effects for most conditions when LPS and S100A8 were added together at 6 h. However, after 24 h, cytokine/chemokine levels released from AECI or AECII were similar irrespective of the stimulus/combination used, which may be due to secretion of the maximum of available cytokines/chemokines by AECs.

We show for the first time that endogenous S100A8/A9 influences PMN migration in BAL in response to DAMP/PAMP exposure, thus confirming the significance of S100A8/A9 in inflammation-related neutrophil immigration. One of the key features of ALI is infiltration of neutrophils into lungs. We demonstrate that S100A8 directly induces neutrophil recruitment into the BAL. Accordingly, S100A9 KO mice showed reduced infiltration of neutrophils into lungs after LPS exposure. This could be explained by the fact that LPS exposure led to a high S100A8/A9 release in BAL of WT mice as compared to the negligible release in S100A9 KO mice. Also, exogenous S100A8 exposure led to higher neutrophil recruitment in BAL of WT mice compared to saline exposure. These facts indicate that LPS induces S100A8/A9 release in BAL, and possibly LPS and S100A8/A9 together activate lung cells like the AECs, which may lead to cytokine/chemokine release and subsequent neutrophil chemotaxis toward lungs. Thus, in this context, local release of S100A8/A9 leads to neutrophil recruitment to lungs which is in line with a previous study showing that the systemic release of S100A8/A9 drives neutrophil adherence and transendothelial recruitment (47). Binding of S100A8/A9 to TLR4-MD-2 on neutrophils activates β2 integrin on neutrophils that in turn binds to ICAM-1 causing neutrophil adherence (47). Our data confirm now also a relevant role of exogenous S100A8/A9 release in BAL of LPS-treated WT mice. No significant differences were found between WT and S100A9 KO mice when PMN counts were made 24 h after LPS exposure. This could be due to secondary or counteracting mechanisms for missing S100A8/A9 release acting in the S100A9 KO mice that replenish PMN counts within 24 h. Although a recent study described that S100A8 helps in reducing inflammation during ALI (48), we observed the opposite. The authors described in their study that the neutrophil immigration to BAL 4 h after S100A8 administration was negligible (48). In contrast, we observed significant neutrophil migration to BAL of WT mice 4 h after S100A8 and LPS treatment as compared to non-treated (saline treated) WT mice. We also observed that the IgM release in BAL 4 h after S100A8 treatment was slightly higher than basal levels (saline treatment), which indicate mild inflammation at 4 h by S100A8 treatment. IgM levels depict the early immune response during an inflammation, which proves that S100A8 is capable of inducing a mild inflammation response within 4 h of administration. We also found that the inflammatory response shown by S100A9 KO mice was significantly lower as compared to the WT mice, as indicated by PMN infiltration upon LPS/S100A8 treatment. Thus, our studies show that S100A8/A9 definitely plays a role in enhancing the inflammation in the context of ALI, in contrast to earlier studies (48, 49) but in line with many other studies (21, 50–52). Our *in vitro* studies also show that S100A8 was capable of generating an inflammatory response, as indicated by high cytokine/chemokine release from AECs and upregulation of TLR4-MD-2 in WT AECII.

We also observed that TLR4 and RAGE did not influence the migration of neutrophils itself *in vivo* upon S100A8 exposure. This observation is supported by a previous study (52), which reported that the phagocyte recruitment to BAL of S100A9 exposed mice was TLR4 and RAGE independent for still unknown reasons. We observed that LPS induced neutrophil recruitment depends on TLR4 as TLR4 KO mice show significantly lower PMN counts compared to WT mice. This is because TLR4 is the main receptor for LPS and thus in the absence of TLR4, LPS induced neutrophil recruitment is hampered. Our observation is supported by a previous study showing that after administration of *Haemophilus influenza* (that contain LPS) in the lungs, TLR4 deficient mice strains had lesser neutrophil counts in the BAL as compared to TLR4 expressing mice strains (53). Although the difference between WT and RAGE KO mice in the context of LPS induced PMN migration is not significant in the current experimental settings, there is an increase in PMN counts in BAL of WT mice as compared to RAGE KO mice. However, this could be explained by release of an endogenous RAGE ligand induced by LPS. HMGB1 may be a candidate which has been shown to be induced by LPS (54, 55). To this end, RAGE has been shown to be involved in causing injury in murine models of experimental ARDS and RAGE blockade was shown to improve arterial oxygenation, alveolar fluid clearance, decreased histological lung injury as evidenced by lesser neutrophil infiltration in lung sections and enhancement of alveolar capillary barrier (56). Effects of S100A8 on neutrophil recruitment were not decreased in this *in vivo* setting, neither in TLR4 nor in RAGE KO mice, indicating a redundant pathway induced by S100A8 in lung tissue *in vivo* which can overcome the lack of TLR4-dependent chemokine induction. Identification of target cells and receptors of this unknown pathway will need a complex approach but will be addressed in a follow-up project.

S100A8/A9 heterocomplexes are released systemically and locally in various infections/injury and contribute to disease severity. High amounts of S100A8/A9 were released in the peritoneal lavage fluid and plasma of peritonitis affected mice, and this lead to liver and lung damage, and high cytokine production, because in S100A9 KO mice, the organ damage was reduced and the cytokine production was diminished (57). Also, elevated levels of S100A8/A9 in plasma of cystic fibrosis patients compared to healthy controls were found and the high levels correlated with high sputum production and C-reactive protein (8). Another study found that in mice exposed to mechanical ventilation/LPS injury, S100A8/A9 deficiency saved mice from lung inflammation, as seen by decreased alveolar-epithelial permeability, low neutrophil influx, and less cytokine/chemokine release in BAL of S100A9 KO mice compared to WT mice (21). Also, high levels of S100A8/A9 have been shown to increase disease severity in mice with induced pneumonia, by aiding in bacterial growth (9).

Our study showed for the first time a TLR4-dependent activation of AECs by S100A8, in the context of cytokine release. Although it was shown previously (19), that S100A8/A9 activates AECs to produce IL8, the receptor involved was not investigated. LPS had been already shown to activate AECII *via* TLR4 (45), but the role of TLR4 in S100A8 mediated AEC activation was unknown till now. Even though a previous study showed that S100A8 and S100A9 induced cytokine production *via* TLR4 in human and murine leukocytes (52), no information was available on the mechanism by which S100A8 or S100A9 induced cytokine production in AECs. Moreover, since previous studies had shown the presence of RAGE majorly in the lungs and particularly in human and rat AECI (44, 58), there was a significant possibility of RAGE being the main receptor involved in AECI activation by S100A8. However, we now have shown evidence that although RAGE is abundantly present in AECI, it does not influence the S100A8 mediated activation of AECI or AECII. Since AECs are one of the first cells to encounter infection or injury in the lungs, therefore activation of AECs may play a major role in enhancing inflammation during ALI. The clinical significance of our study lies in identifying the underlying cause behind this activation, i.e., the interaction between S100A8 and TLR4 in AECs. Therefore, interrupting the binding of S100A8 to TLR4 by targeting S100A8 residues involved in TLR4 binding could be a therapeutic option in ALI and other inflammatory lung diseases.

## Ethics Statement

This study was carried out in accordance with the recommendations of the local ethics committee and performed in strict accordance with the German regulations of the Society for Laboratory Animal Science (GV-SOLAS) and the European Health Law of the Federation of Laboratory Animal Science Associations (FELASA). The protocols were approved by the Landesamt für Natur, Umwelt und Verbraucherschutz Nordrhein-Westfalen (LANUV-NRW), Germany.

## Author Contributions

DC contributed to the study conception, experimental design, data acquisition, data analysis and interpretation, writing, and revision of the manuscript. SZ contributed to the experimental design, data analysis and data interpretation. AH contributed to data acquisition for the revision of the manuscript. JRossaint contributed to the experimental design, data acquisition, and analysis of *in vivo* experiments. AH contributed to data acquisition for the revision of the manuscript. MP contributed to the data acquisition. AZ contributed to the conception and experimental design of *in vivo* experiments. JRoth contributed to the study conception, data interpretation, writing, and revision of the manuscript. TV contributed to the study conception, experimental design, data interpretation, writing, and revision of the manuscript.

## Conflict of Interest Statement

The authors declare that the research was conducted in the absence of any commercial or financial relationships that could be construed as a potential conflict of interest.

## Supplementary Material

Figure S1 Characterization of the purification process and culture of alveolar epithelial cells (AECs). **(A)** Cells were stained at the end of the purification process by flow cytometry for leukocyte markers like CD45, CD16-32, and CD34. Solid histograms depict isotype controls, open histograms depict CD45/CD16-32/CD34 staining. **(B)** AECs were grown in DMEM with 10% FBS [DMEM (10% FBS)] or in airway epithelial media (without FBS) for 3 days and the media collected after 3 days was analyzed for IL-6 secretion by enzyme-linked immunosorbent assay. **(C)** Primary AECs were stained for pro-SPC (FITC) immediately after isolation by flow cytometry. Solid histogram depicts isotype control, open histogram depicts pro-SPC staining. proSP-C = Prosurfactant protein C, FITC = Fluorescein isothiocyanate.

Figure S2 Stimulation of human monocytes and AECI with lipopolysaccharide (LPS) or S100A8. **(A)** Human monocytes were stimulated with different concentrations of LPS, or murine S100A8 (5 µg/ml) in presence and absence of Polymyxin B (25 µg/ml) for 4 h. TNF-α release was measured by enzyme-linked immunosorbent assay (ELISA). **(B)** Confluent monolayers of AECI were stimulated for 6 h with different concentrations of LPS or murine S100A8 (5 µg/ml). Polymyxin B (25 µg/ml) was added in combination with LPS (10 ng/ml) or S100A8 (5 µg/ml). IL-6 release was measured by ELISA.

Figure S3 TLR4-MD-2 expression in AECII stimulated by murine S100A8 or lipopolysaccharide (LPS) in presence of Polymyxin B. WT AECII were cultured for 1 day, and then stimulated by murine S100A8 (5 µg/ml) or LPS (10 ng/ml) alone or in combination with Polymyxin B (25 µg/ml) for 6 h, followed by staining for TLR4-MD-2 (Streptavidin Dylight 488). Nuclei were stained with DAPI. Imaging was performed by an inverted fluorescent microscope (Axio Observer, Zeiss), *n* = 3 experiments and a representative image is shown. Scale bar is 50 µm.

Figure S4 Western blot of bone marrow cells of WT and S100A9 KO mice. Bone marrow cells were isolated from three WT and S100A9 KO mice and lysed for western blot staining. Rabbit polyclonal S100A8 and rabbit polyclonal S100A9 antibodies were used for the staining. β actin was used as a positive control for western blot. Imaging was performed by a ChemiDoc Gel Imaging system.
