# Supplementary figures and images for: Alarmin S100A8 Activates Alveolar Epithelial Cells in the Context of Acute Lung Injury in a TLR4-Dependent Manner
Source: Front Immunol. 2017 Nov 13;8:1493. doi: 10.3389/fimmu.2017.01493 (PMC5693860; doi:10.3389/fimmu.2017.01493)

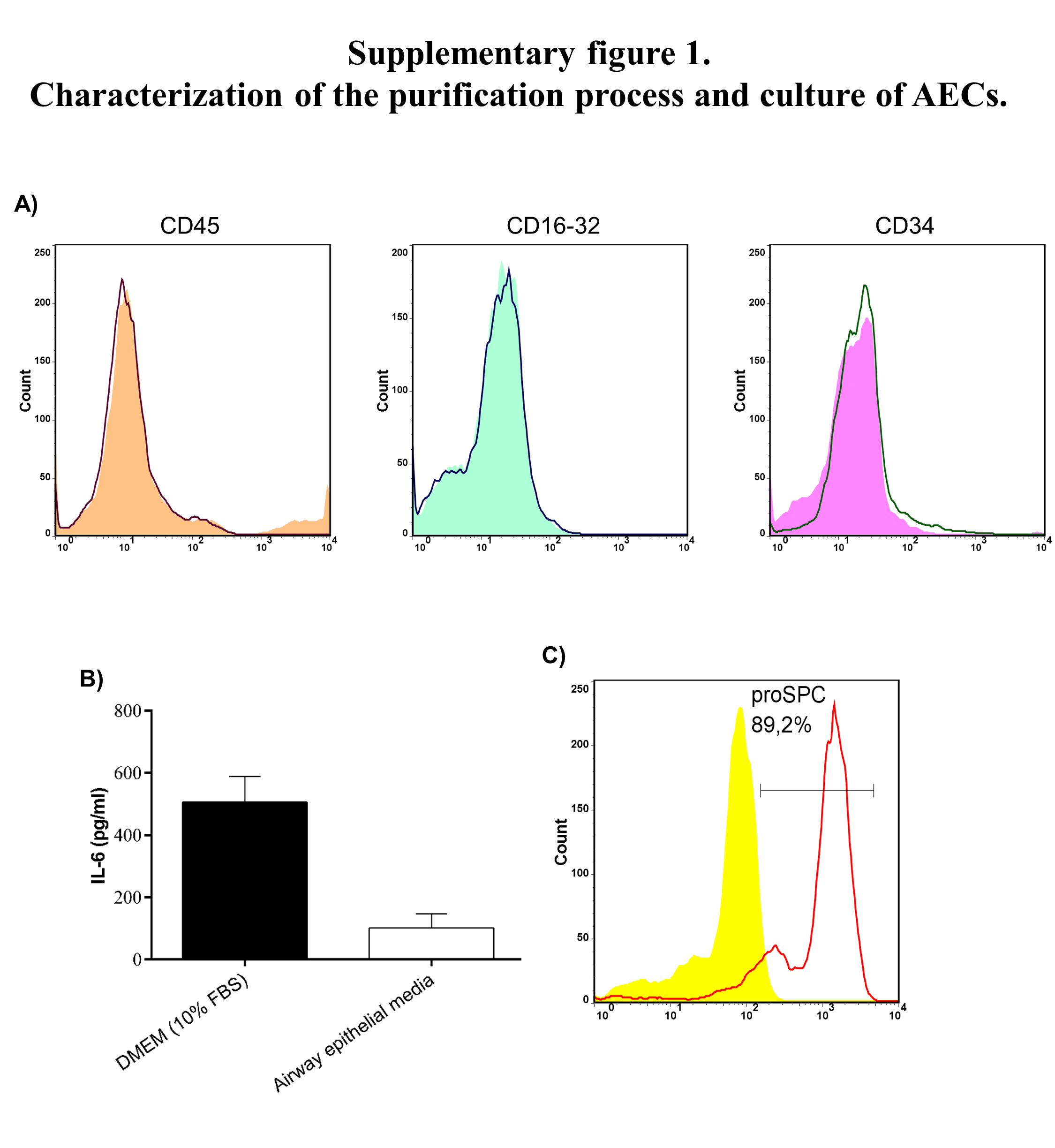

Supplement: Figure S1 — Characterization of the purification process and culture of alveolar epithelial cells (AECs). (A) Cells were stained at the end of the purification process by flow cytometry for leukocyte markers like CD45, CD16-32, and CD34. Solid histograms depict isotype controls, open histograms depict CD45/CD16-32/CD34 staining. (B) AECs were grown in DMEM with 10% FBS [DMEM (10% FBS)] or in airway epithelial media (without FBS) for 3 days and the media collected after 3 days was analyzed for IL-6 secretion by enzyme-linked immunosorbent assay. (C) Primary AECs were stained for pro-SPC (FITC) immediately after isolation by flow cytometry. Solid histogram depicts isotype control, open histogram depicts pro-SPC staining. proSP-C = Prosurfactant protein C, FITC = Fluorescein isothiocyanate. [file Image_1.TIF]

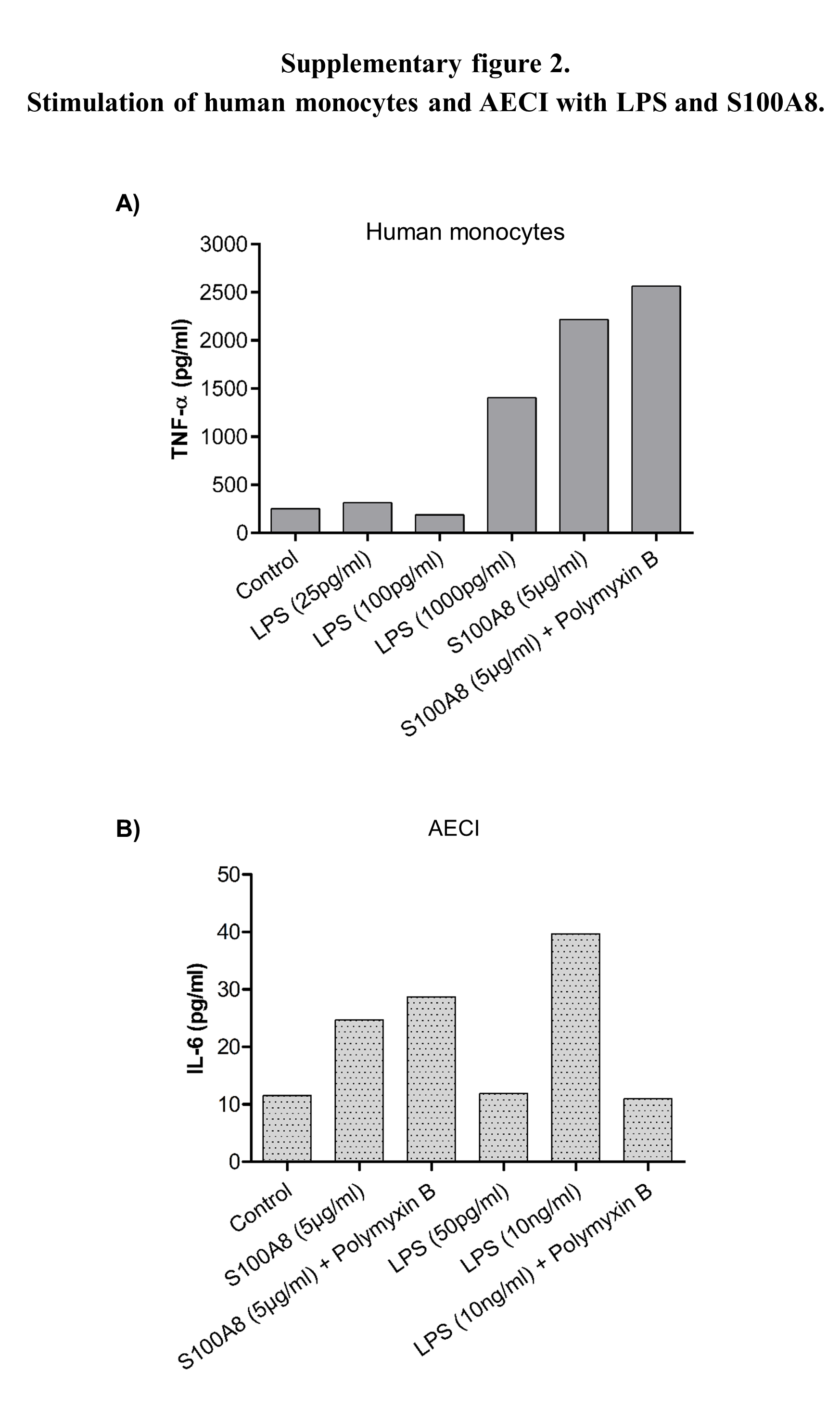

Supplement: Figure S2 — Stimulation of human monocytes and AECI with lipopolysaccharide (LPS) or S100A8. (A) Human monocytes were stimulated with different concentrations of LPS, or murine S100A8 (5 µg/ml) in presence and absence of Polymyxin B (25 µg/ml) for 4 h. TNF-α release was measured by enzyme-linked immunosorbent assay (ELISA). (B) Confluent monolayers of AECI were stimulated for 6 h with different concentrations of LPS or murine S100A8 (5 µg/ml). Polymyxin B (25 µg/ml) was added in combination with LPS (10 ng/ml) or S100A8 (5 µg/ml). IL-6 release was measured by ELISA. [file Image_2.TIF]

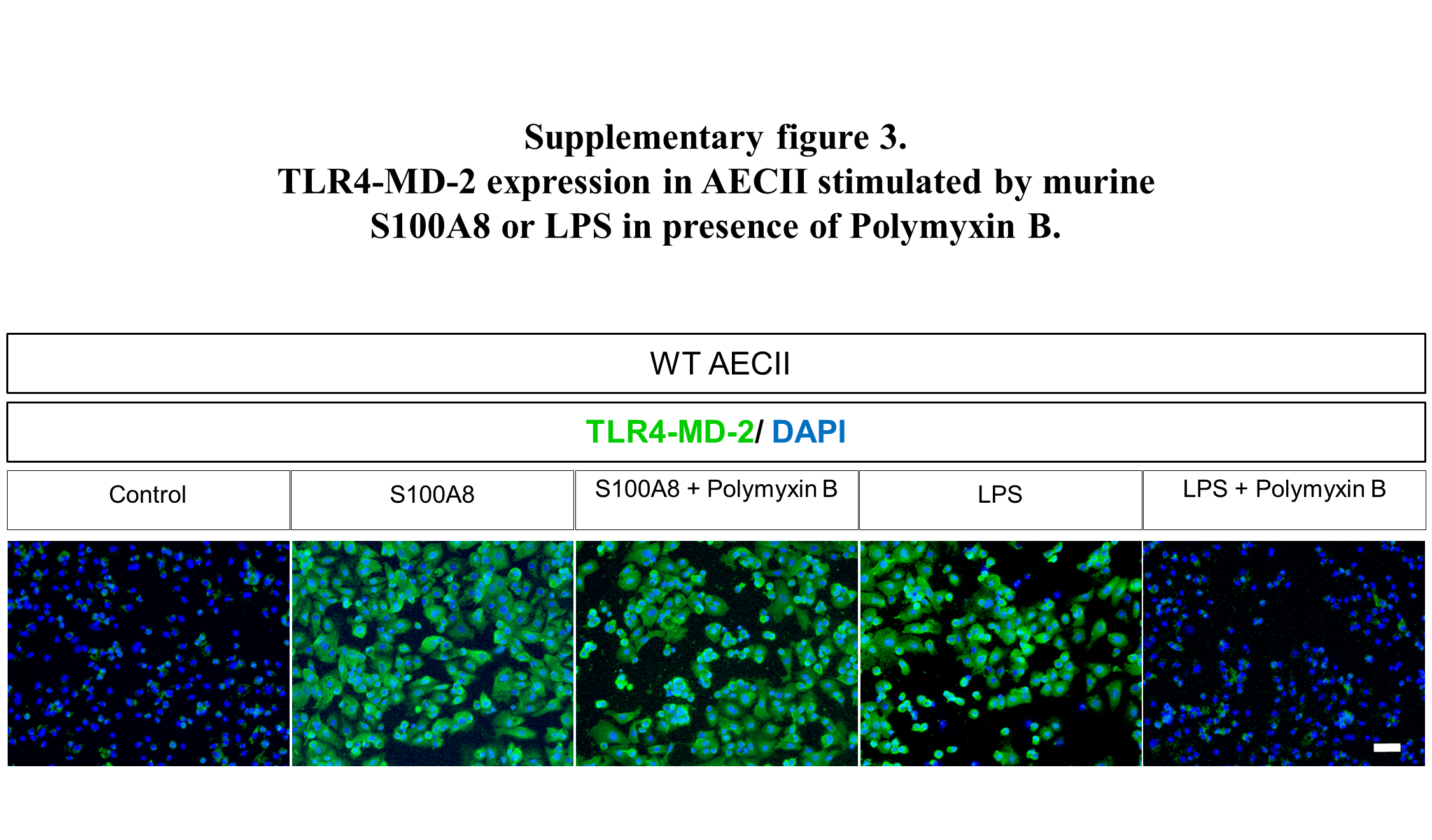

Supplement: Figure S3 — TLR4-MD-2 expression in AECII stimulated by murine S100A8 or lipopolysaccharide (LPS) in presence of Polymyxin B. WT AECII were cultured for 1 day, and then stimulated by murine S100A8 (5 µg/ml) or LPS (10 ng/ml) alone or in combination with Polymyxin B (25 µg/ml) for 6 h, followed by staining for TLR4-MD-2 (Streptavidin Dylight 488). Nuclei were stained with DAPI. Imaging was performed by an inverted fluorescent microscope (Axio Observer, Zeiss), n = 3 experiments and a representative image is shown. Scale bar is 50 µm. [file Image_3.TIF]

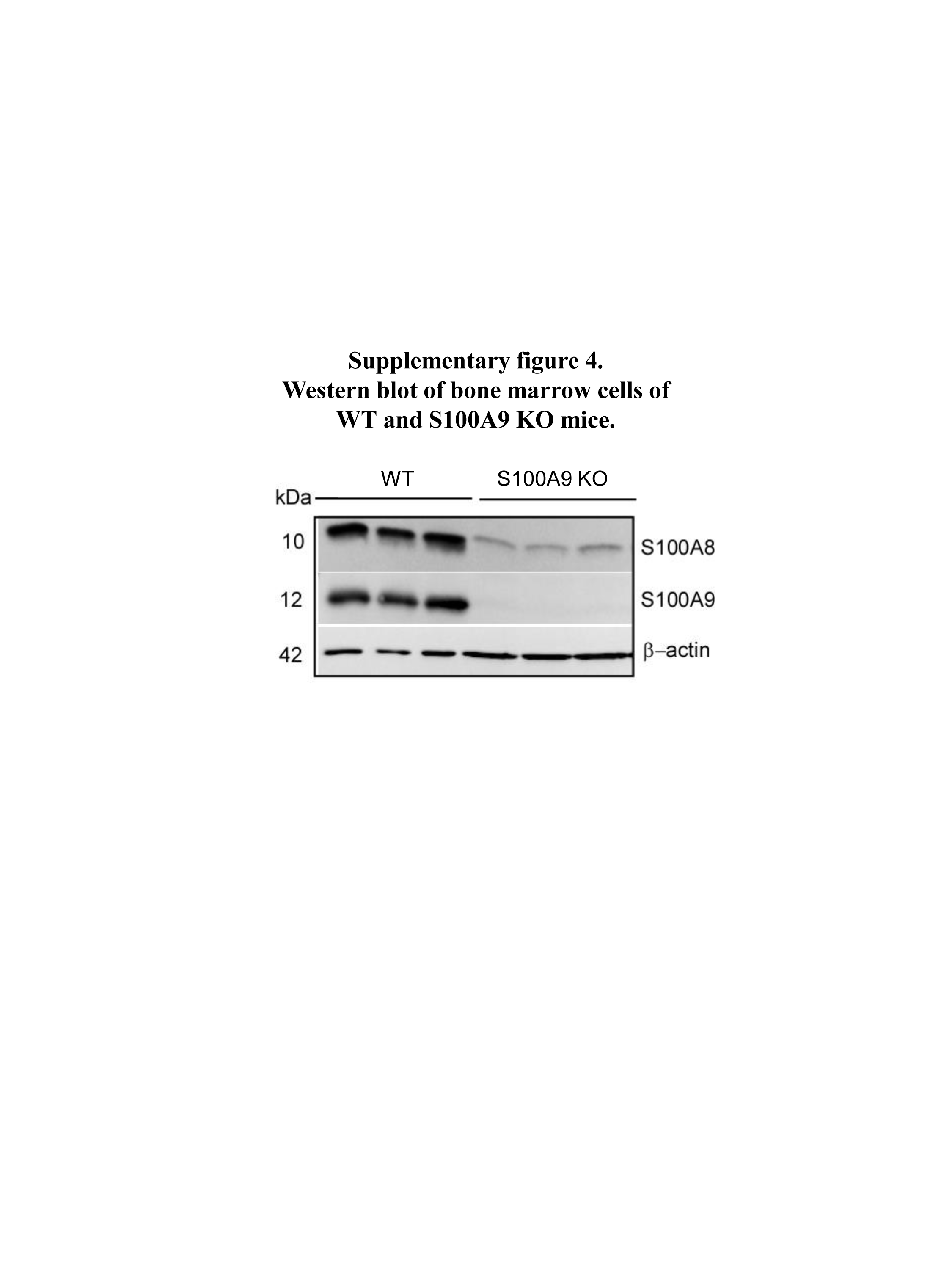

Supplement: Figure S4 — Western blot of bone marrow cells of WT and S100A9 KO mice. Bone marrow cells were isolated from three WT and S100A9 KO mice and lysed for western blot staining. Rabbit polyclonal S100A8 and rabbit polyclonal S100A9 antibodies were used for the staining. β actin was used as a positive control for western blot. Imaging was performed by a ChemiDoc Gel Imaging system. [file Image_4.TIF]
